# A novel naturally superoleophilic coconut oil-based foam with inherent hydrophobic properties for oil and grease sorption

**DOI:** 10.1038/s41598-024-64178-2

**Published:** 2024-06-20

**Authors:** Tomas Ralph B. Tomon, Christine Joy M. Omisol, Blessy Joy M. Aguinid, Karyl Xyrra L. Sabulbero, Arnold C. Alguno, Roberto M. Malaluan, Arnold A. Lubguban

**Affiliations:** 1 Center for Sustainable Polymers, Mindanao State University – Iligan Institute of Technology, 9200 Iligan City, Philippines; 2 Department of Physics, Mindanao State University – Iligan Institute of Technology, 9200 Iligan City, Philippines; 3 Department of Chemical Engineering and Technology, Mindanao State University – Iligan Institute of Technology, 9200 Iligan City, Philippines

**Keywords:** Environmental sciences, Chemistry, Materials science, Chemical engineering

## Abstract

Absorption methods using polyurethane foams (PUFs) have recently gained popularity in treating oil spills. However, conventional petroleum-based PUFs lack selectivity and are commonly surface-modified using complicated processes that require toxic and harmful solvents to enhance their hydrophobicity and oil sorption capacities. In this paper, a novel naturally superoleophilic foam with inherent hydrophobic properties has been developed through the conventional one-shot foaming method with the integration of coconut oil-based polyol. This bio-based polyol was explicitly handpicked as it is chiefly saturated, highly abundant, and inexpensive. The foam is characterized by an oil sorption capacity range of 14.89–24.65 g g^−1^ for different types of oil, equivalent to 578–871 times its weight. Its hydrophobic behavior is expressed through a water contact angle of ~ 139°. The foam also showcased excellent chemical stability and high recyclability without a significant loss in absorption capacity after 20 cycles. The incorporation of the coconut oil-based polyol is also shown to improve the morphological, mechanical, and thermal behavior of the foam. It can be inferred from these findings that this novel material holds great potential for revolutionizing sorbents, pioneering a more sustainable and eco-friendly functional material produced via a facile method.

## Introduction

The degradation of the world’s oceans caused by oil pollution is a pressing matter. Oil spills and oily industrial wastewater are among the primary drivers of oil pollution in the ocean^1–5^. Its widespread catastrophic effects on the environment, human health, aquatic life, and the economy are evidenced by the disruption of marine and coastal ecosystems, water, land, and air pollution, health complications, and wreaked havoc to various economic sectors, such as the fishing, food, and tourism industries, to name a few^6^. Over time, various chemical, mechanical, and biological techniques have been used to remove and recover oils from water^7–9^. However, methods from these categories suffer drawbacks due to their high production cost and low efficiency, among other things^7^. The absorption technique, on the other hand, is highly regarded because of its cost-effectiveness^10^, efficiency^11^, and excellent reusability^12^, which are deemed ideal in choosing the proper treatment method. Therefore, significant efforts have been instituted to develop low-cost and practical sorbents that can potentially be used for oil spill clean-up operations.

Various types of natural and synthetic sorbents in both two-dimensional (2D) and three-dimensional (3D) forms, like fabrics, fibers, membranes, meshes, sponges, foams, and nanoparticles, were developed to aid in separating oil from water^13–17^. Out of these, 3D sorbents are more desirable due to their large surface area, excellent recyclability, and highly porous nature, which allows for greater storage capacity. Polyurethane foams (PUFs), with their distinctive 3D structure, have a broad range of applications, one being a sorbent material commonly used in wastewater treatment^18^ owing to their high porosity and surface area, availability, and versatility. However, these foams lack selectivity between oil and water. When utilized for oil spill cleanup, they also absorb large amounts of water, thereby limiting their performance and efficiency^3^. Recent breakthroughs have addressed this limitation via surface modification. However, this technique often uses harmful solvents and complex procedures^3,19^, which warrants higher costs and additional resources. Moreover, the conventional manufacturing process of PUFs employs petroleum-based polyols, rendering it even more ruinous to the environment^18^. As a result, it is imperative to devise a greener alternative of hydrophobic sorbent for oil spill clean-up efforts that is facile, cost-efficient, and environmentally friendly.

In the present study, a bio-based polyol derived from coconut oil was used to synthesize a highly recyclable novel naturally superoleophilic foam (NSF) with inherent hydrophobic properties. The predominantly saturated nature of coconut oil^20^ and the recent development in its suitability for PUF synthesis^21^ motivated the notion of exploring the latent qualities of coconut oil-based PUF as an oil sorbent. Fourier transform infrared spectrometer (FTIR), thermogravimetric analyzer (TGA), scanning electron microscope (SEM) and pycnometer, and universal testing machine (UTM) were used to characterize the functional, thermal, morphological, and mechanical properties of NSF, respectively. These properties have been examined in comparison with a control petroleum-based PUF to determine the effects of polyol bio-replacement. Finally, the sorption performance of NSF was quantitatively evaluated in terms of contact angle, sorption capacity, recyclability, and chemical stability.

## Materials and methods

### Materials

The synthesis of NSF involves the usage of a polyol consisting of a blend of bio-based and petroleum-based polyols. The coconut oil used to synthesize the bio-based polyol component of the blend was purchased from a local store. The petroleum-based polyol (Voranol^®^ 4701, polyether polyol), glycerol, dispersant silicone oil, and polymeric methylene diphenyl diisocyanate (pMDI, PAPI 135 SH) were provided by Chemrez Technologies, Inc. (Quezon City, Philippines). The catalysts (Polycat^®^ 8, Polycat 5, calcium oxide (CaO), and stannous octoate), and the phthalic anhydride (PA, AR-grade) used in this study were obtained from Sigma-Aldrich Chemicals, Philippines. The contaminants examined in this study, such as vegetable oil, engine oil, and used engine oil, were commercially acquired, while the bunker fuel was kindly provided by Mabuhay Vinyl Corporation (Iligan City, Philippines). All materials used in this study were used as is and without any modification.

### Synthesis of the bio-based polyol

The bio-based polyol derived from coconut oil used in this study was developed systematically in a two-step method. A pre-determined amount of coconut oil, glycerol, and CaO was stirred continuously in a closed Parr reactor at 250 °C for 90 min to break down coconut triglycerides and produce monoglycerides. The resulting product was allowed to cool down before subjecting to the second step that was employed in a previous study^21^ to produce a coconut oil-based polyol.

### Synthesis of the sorbent foam

The NSF was produced through a free-rise method that involved mixing the A-side (pMDI) and B-side (polyol mixture) components. To evaluate the effect of the synthesized bio-based polyol on foam properties, 50 wt% of the polyol part was replaced. The synthesis of the foam was initialized by premixing the B-side components, containing the stoichiometric mixture of bio-based polyol, water, surfactants, and catalysts in a cup mold at 2000 rpm for 60 s to ensure homogeneity. Then, a stoichiometrically calculated amount of pMDI was added to the polyol pre-mix and stirred vigorously at the same speed for 5–10 s. The resulting mixture was undisturbed, allowing it to expand freely on the mold. It was then left to cool and cure at ambient conditions overnight. The same procedure was followed using 100% petroleum-based polyol to synthesize the control sample.

### Determination of the oil/water sorption capacity

Different types of oils with varying viscosities, including vegetable oil, engine oil, used engine oil, and bunker fuel, were tested along with distilled water and seawater to showcase the effectiveness of the NSF in absorbing oil while resisting water. The sorbate was poured into a 100-mL beaker, ready for the sorption test. The NSF sample was initially weighed using an analytical balance before testing, where the foam was dropped on top of the sorbate and allowed to perform sorption. After 10 min of contact, the NSF was recovered from the setup using forceps and was allowed to drain for 10 ± 3 s to remove any excess oil or water. The saturated NSF was then weighed and recorded, and the test was repeated. The sorption capacity of the sorbent material was determined using Eq. (1):

1 $$Q = \frac{{m_{f} - m_{0} }}{{m_{0} }} \times 100$$

where *Q* is the oil sorption capacity (g g^−1^), *m*_*f*_ is the weight of the foam (g) after the sorption test, and *m*_0_ is the initial weight of the foam (g).

### Recyclability test

To evaluate the recyclability of the NSF sorbent, a simple absorption–desorption process was employed. This test follows the sorption capacity test for oil and water. Following this, the saturated foam was centrifuged at 1000 rpm for 60 s to remove any excess oils. This technique allows for the efficient and reliable determination of the absorption efficiency of the foam, which can be used to optimize and improve the foam's recyclability for future applications. The absorption efficiency of the NSF was calculated using Eq. (2):

2 $${Q}_{eff}(\%)= \frac{{w}_{abs}}{{w}_{0}} \times 100$$

where *Q*_*eff*_ is the absorption efficiency (%), *w*_*abs*_ is the *nth* mass of the foam (g) after soaking, and *w*_*0*_ is the initial weight (g) of the foam after soaking.

### Chemical stability test

The stability of the foams was assessed by subjecting them to simulations in acidic, saline, and basic environments. Each foam was immersed in 30 mL of a solution containing HCl (2 M, pH 0), a concentrated solution of NaCl (26.5% by weight, pH 7), and NaOH (2 M, pH 14) for 24 h. The initial and final weight, tensile strength, and microstructure were recorded to account for these changes as a measure of the material’s chemical resistance and stability according to ASTM D543^22^. The weight changes of the foams were calculated using Eq. (3)^2,23^:

3 $$\% weight\;retained=\frac{{w}_{0}- {w}_{f}}{{w}_{0}} \times 100$$

where *w*_*0*_ is the initial weight (g), and *w*_*f*_ is the final weight of the foam (g) after the stability tests, and were performed in triplicate.

### Open-cell content determination

The masses and dimensions of the foams were measured to calculate their apparent densities. The open-cell content of the foam was determined by using a pycnometer (Quantachrome ULTRAPYC 1200e, FL USA) and was subsequently calculated using Eq. (4)^24^:

4 $$OC \left( \% \right) = \frac{{v_{g} - v_{p} }}{{v_{g} }} \times 100$$

where *OC* is the open-cell content of the sample foam, *v*_*g*_ is the geometric volume, and *v*_*p*_ is the pycnometric volume.

### Characterization

To study the functional groups and the surface morphology of the synthesized NSF, the samples were characterized using FTIR (Shimadzu ATR-FTIR IRTracer-100, Kyoto, JPN) and SEM (JEOL JSM-IT200, Tokyo, JPN), respectively. The thermal degradation profile was analyzed using TGA (PerkinElmer TGA 4000, Waltham, MA) under a nitrogen atmosphere (flow rate 20 mL min^−1^) and a heating rate of 10 °C min^−1^. The universal testing machine (UTM, Shimadzu AGS-XSeries, Kyoto, JPN) was used to test the mechanical and physical properties of the foam according to ASTM standards. The water contact angle (WCA) measurements were conducted at ambient conditions using an optical tensiometer (Biolin Scientific ThetaLite 101, Gothenburg, Sweden).

## Results and discussion

### Chemical analysis of the NSF sorbent

**Figure 1:**
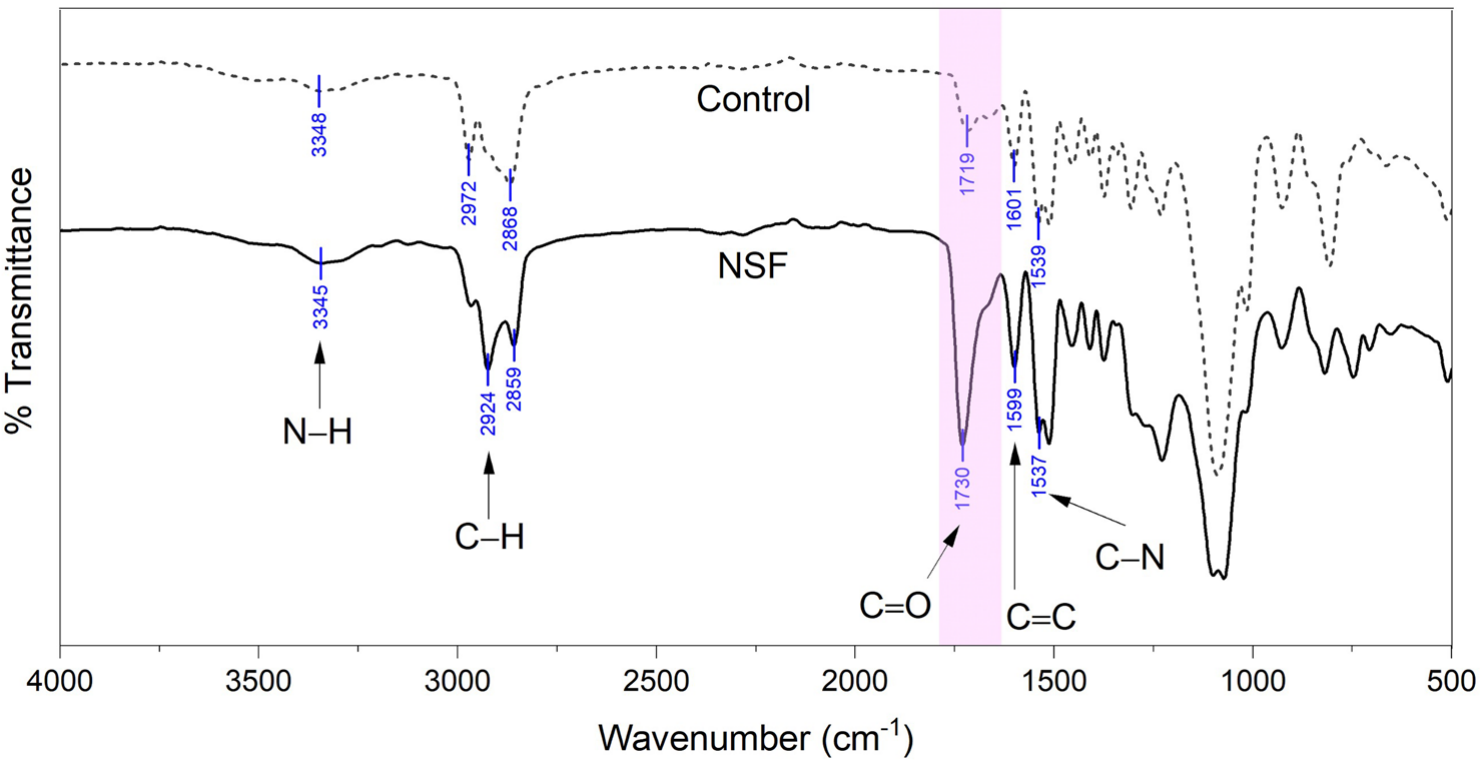
FTIR spectra comparison of a pristine petroleum-based polyurethane foam (control) against the synthesized natural superoleophilic foam (NSF).

The FTIR spectra of the foams shown in Fig. 1 depict differences in the key transmission bands of NSF and the control PU. The peak at 3345 cm^−1^ indicates the N–H stretching vibrations of the urethane bonds. This peak at the NSF spectrum is distinctively broader and more pronounced than the equivalent peak of the control PU at 3348 cm^−1^, signifying the presence of excess OH groups^23^, a general characteristic of flexible foams. The weak bands at 2924 cm^−1^ and 2859 cm^−1^ on the NSF spectrum represent the stretching vibrations of the C–H groups. These peaks have greater intensity than the corresponding peaks on the control PU at 2972 cm^−1^ and 2868 cm^−1^, signifying the presence of fatty acid chains in the coconut oil-based polyol^25^. The peak at 1730 cm^−1^ for NSF and 1719 cm^−1^ for the control PU denotes the stretching of the C=O bond in the PU structure. Moreover, the stretching band at 1599 cm^−1^ for NSF and 1601 cm^−1^ for the control PU shows the aromatic C=C groups. These peaks have conspicuously augmented peaks for NSF, indicative of the successful incorporation of the ester- and phenyl-rich coconut oil-based polyol in PU structure. Similarly, the stretching vibration band of C–N at 1537 cm^−1^ on the spectrum of NSF also has an increased intensity than the peak on the control at 1539 cm^−1^ owing to the higher OH number of the coconut oil-based polyol, thus forming more urethane linkages^21^.

### Thermal analysis

**Figure 2:**
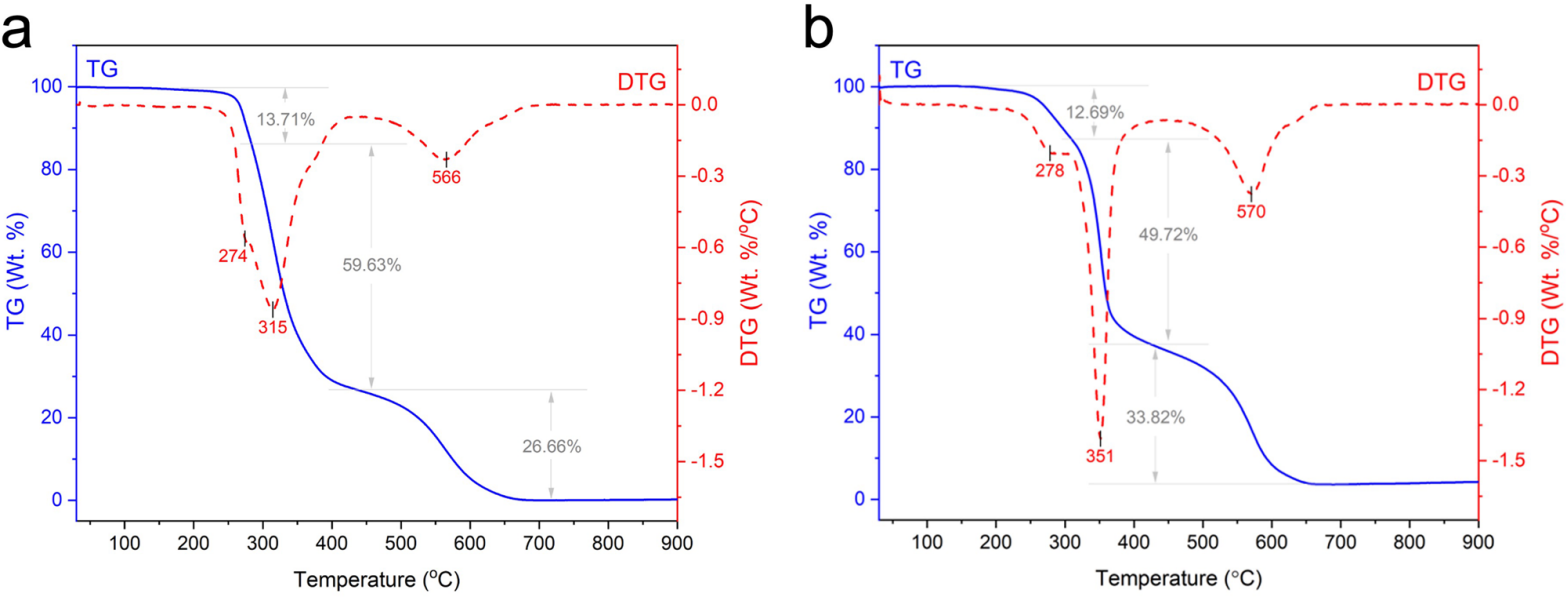
TG and DTG curves of the (**a**) control polyurethane foam and (**b**) naturally superoleophilic foam (NSF).

Thermogravimetric analysis was deployed to determine the thermal stability of the foams by examining their weight loss as a function of temperature. Figure 2 depicts the TG and DTG curves of the samples. It can be observed that both TG and DTG thermographs of the foam samples reveal a similar thermal behavior with three distinct degradation peaks. The first stage of degradation is observed to occur at around 274 °C for the control PU foam and 278 °C for NSF, owing to the decomposition of urethane linkages^21,26,27^. A striking difference in the foams’ thermal stability can be observed at the second stage of decomposition, assigned to the degradation of soft segments^28^. This occurred at 315 °C for the control foam and 351 °C for NSF. The distinctive difference between the soft segment degradation of the control and NSF can be ascribed to the coconut oil-based polyol component in NSF being a polyester. Polyester polyols are generally more thermally stable than polyether polyols^29^. Thus, its consolidation into the polyol system and, subsequently, into the PU matrix enhanced the overall thermal stability of the resulting NSF. The third and final stage of degradation at 566 °C for the control PU and 570 °C for NSF represents the degradation of the isocyanate (NCO) groups^21,27^. It is evident from Fig. 2 that the weight loss in NSF at this stage is greater than the control. This difference is linked to the increased NCO requirement of the coconut oil-based polyol due to its high OH number. These findings suggest that the bio-replacement of the petroleum-based polyol with coconut oil-based polyol improved the thermal stability of the resulting PU foam^30^.

### SEM and pycnometric analysis

**Figure 3:**
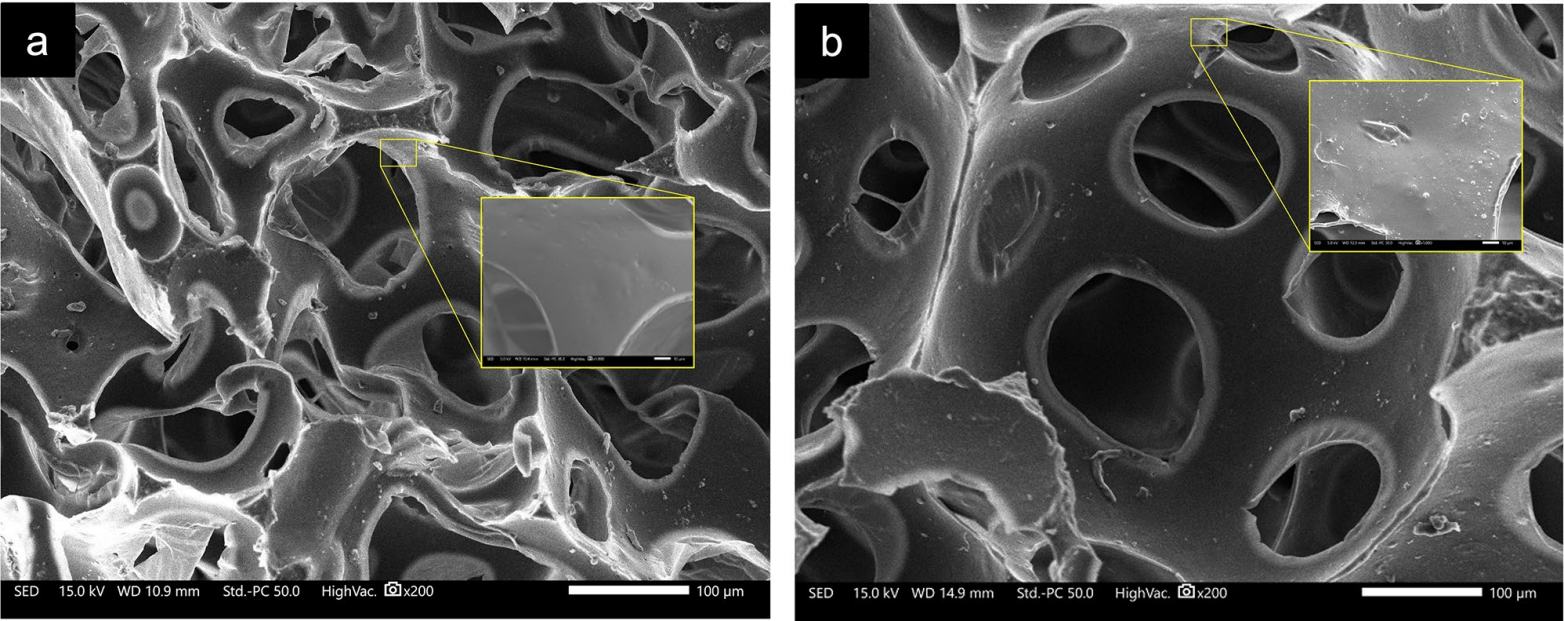
Scanning electron microscope (SEM) images of the (**a**) control polyurethane foam and (**b**) naturally superoleophilic foam (NSF) at ×200 with ×1000 magnification.

The images presented in Fig. 3 showcase the SEM micrographs of the control PU and the NSF. The microstructure of control PU depicted in Fig. 3a reveals a continuous and smooth surface characterized by large open cells with irregular shapes. Conversely, Fig. 3b illustrates the microstructure of a macro-porous NSF. It can be observed that NSF shows a visible demonstration of a more homogeneous and distinct cell structure. This structural improvement is of great significance as it directly influences the sorption capacity of the foam by enhancing its ability to store oil effectively. Additionally, Fig. 3b displays that the developed NSF exhibited a rougher surface compared with the control PU in Fig. 3a. Increased surface roughness is responsible for trapping air pockets on the surface of the foam, allowing it to repel water by acting as the hydrophobic media^3,7,31^. In this case, the roughening of the surface of NSF may be attributed to the greater crosslinking density of the PU foam matrix due to the higher OH number of the coconut oil-based polyol^21,32^.

Although the microstructures of the foam samples are vastly different, the variance in their average open-cell content is negligible. The average open-cell content of the control and the NSF were 97.16% and 97.21%, respectively, indicating that the incorporation of the coconut oil-based polyol did not have a significant effect on its open-cell content but rather enhanced its cell structure and surface roughness.

### Mechanical strength

**Figure 4:**
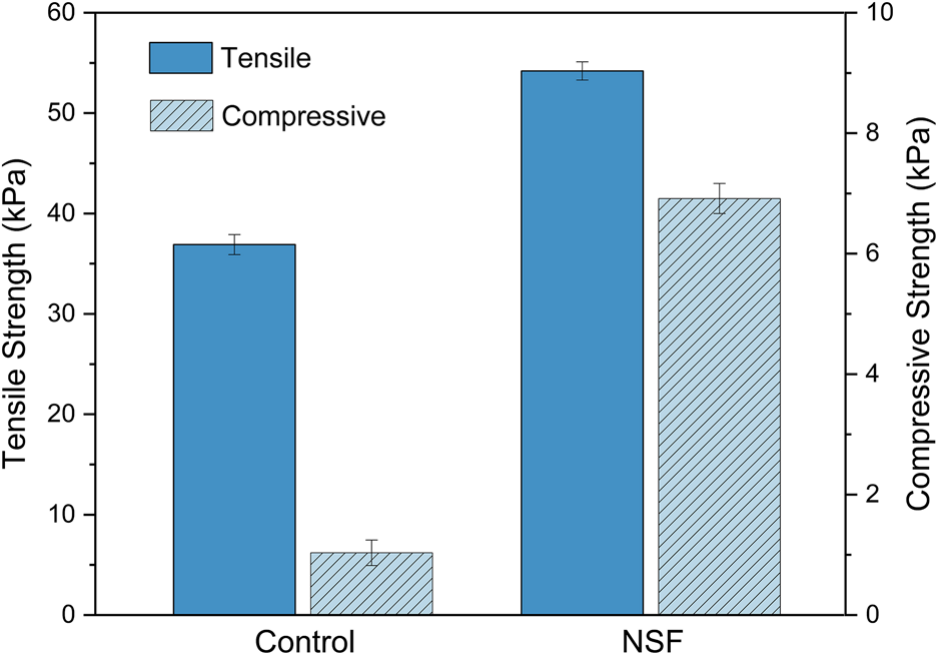
The tensile and compressive strengths of the control polyurethane and the naturally superoleophilic foams (NSF).

Determining the mechanical strength of NSF plays a significant role in dictating the suitability for practical applications in oil spills. Tensile strength and compressive force deflection (CFD) at 50% compression tests were performed on NSF and the control to ascertain the effect of the coconut oil-based polyol on said foam properties. This investigation is especially important since bio-replacement is widely known to have adverse effects on the mechanical properties of PU foams^33^. Figure 4 displays the mechanical performance of the foams.

As is evident in Fig. 4, the bio-replacement of the foam formulation showed a notable increase in the tensile strength of the NSF compared with the control foam, boosting its strength by ~ 47%. Comparably, its compressive strength also increased. The upswing in tensile and compressive behavior was mainly contributed by the increased NCO loading in NSF, which accounted for the higher OH groups in the coconut oil-based polyol. This tends to increase the urethane linkages in the foam matrix, and, to some extent, the residual NCO groups can proceed in secondary reactions with the main reaction products, thus generating stronger linkages that can endure greater tension and compression stresses^34^. Moreover, the presence of sharper N–H peaks in the FTIR spectrum of NSF in Fig. 1 suggests a higher likelihood of hydrogen bonding with other functional groups in the foam matrix, further strengthening its structure. Equally significant is the reinforcing effect of the homogenized three-dimensional cell wall structure of NSF^27^, featured in the SEM image in Fig. 3b. The increased homogeneity in the cell structure and size of the NSF compared with the control foam (Fig. 3a) provides better loading distribution by effectively dispersing applied mechanical stresses, thus increasing supporting strength^27,35^ and making the NSF suitable for practical oil spill applications^27,34,35^.

### Wettability test

**Figure 5:**
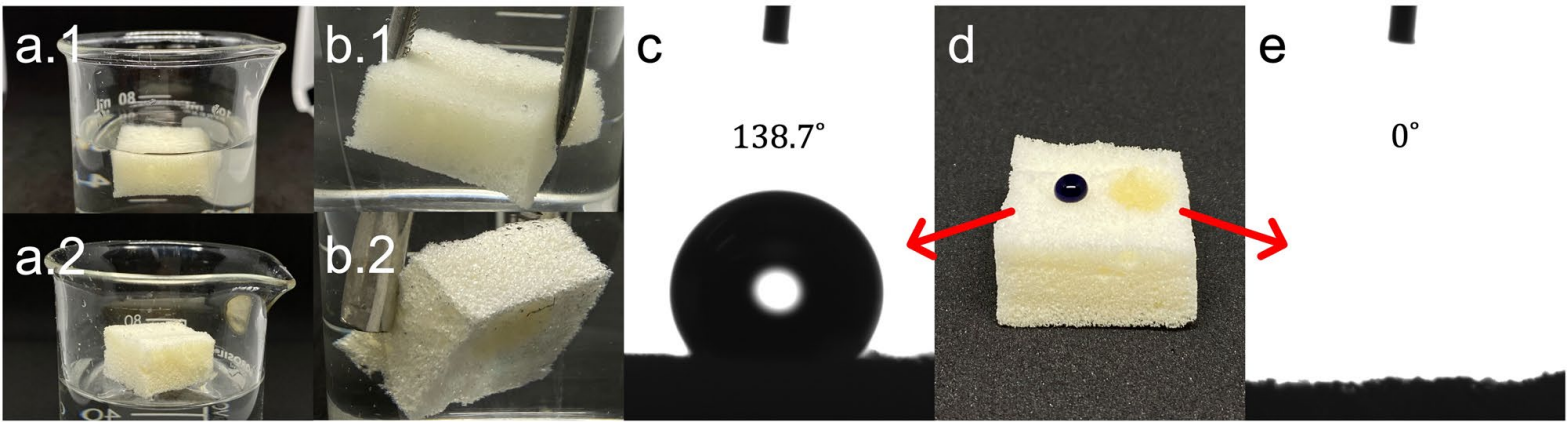
Illustration of the (**a**) floatability and (**b**) plunging tests of the (1) control and (2) naturally superoleophilic foam (NSF) samples, including photographs of the NSF’s (**c,d**) water (dyed blue for visibility), and (**d,e**) oil contact angles.

Figure 5 highlights the crucial role of the contact angle (CA), θ, in understanding the surface-wetting behavior of the foam. This measurement indicates the degree of wettability exhibited by a liquid on a solid surface. Based on the WCA value, three distinct surface property categories can be established: hydrophilic (θ < 90°), hydrophobic (90° < θ < 150°), and superhydrophobic (θ > 150°)^36,37^. These classifications serve as a valuable framework for characterizing and differentiating materials according to their interaction with liquid droplets. The resulting CA of the NSF was obtained through the built-in image analyzer of the optical tensiometer.

Figure 5a and b showcase the remarkable difference in the hydrophobicity of the control and NSF samples through the floatability and plunging tests. The high affinity of PUFs in absorbing water is exemplified by the floatability (Fig. 5a.1) and plunging (Fig. 5b.1) performance of the control, wherein the foam sample immediately submerged underwater and showed no indication of water repellence. Contrastingly, the water-resistant nature of NSF is evident in Figs. 5a.2 and b.2 as the foam effectively floats on top of the water surface and exhibits a silver mirror-like layer at the supposed foam-water interface when forcefully immersed in water. These observations were validated by the NSF’s high WCA of ~ 139° shown in Fig. 5c, classifying it as a hydrophobic material. This lack of affinity for water can be ascribed to the presence of primarily saturated fatty acid side chains on the coconut oil-based polyol in addition to the rough surface morphology of the foam that readily entraps air bubbles, preventing water from permeating into the material. These features work complementarily, resulting in the water-repelling characteristics of the foam and posing as a potentially revolutionary material for practical and comprehensive applications.

Moreover, the wettability of non-polar liquids is also classified into three distinct categories: oleophobic (θ > 90°), oleophilic (0° < θ < 90°), and superoleophilic (θ = 0°)^38,39^. Figure 5e provides cogent visual evidence of the inherent oil sorption capacity of the NSF. In less than 5 s, the foam was able to rapidly absorb the oil droplet, revealing a CA of 0°, thus distinguishing the NSF as superoleophilic. This demonstrates the efficiency of NSF in absorbing oil and highlighting its potential in various oil remediation applications.

### Absorption capacity test of NSF

**Figure 6:**
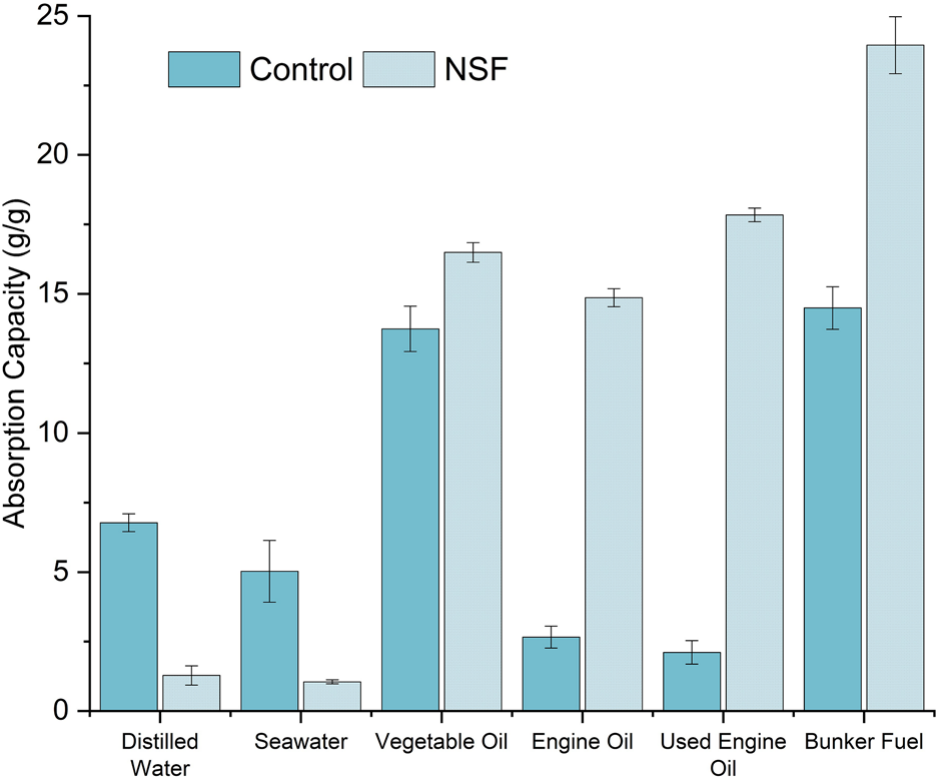
Absorption capacities (g/g) of the control foam and the naturally superoleophilic foam (NSF).

In this study, the foam samples were cut into an approximate dimension of 1 × 1 × 0.5 in and then tested to determine their sorption capacity for water and oil. The sorption capacities obtained for both water and oil were tested in triplicates, and the average values are reported in Fig. 6. The average sorption capacity of NSF is defined as the mass or volume of the sorbate (water or oil) per unit mass or volume of the sorbent. It can be inferred that the NSF can absorb different types of oil ranging between 14.86–23.95 times its weight while only absorbing water at 1.05–1.28 times its weight. In contrast, the control foam is limited to absorbing the oil sorbates only at a range of 2.11–14.49 times its weight and is significantly more absorbent in water with a capacity of 5.03–6.78 times its weight. In terms of volume ratio, the average sorption capacity of NSF revealed a minimal water and seawater uptake equivalent to 4–6% of the sorbent’s volume, calculated using the density of the sorbates in Table 1. In comparison, the NSF showed greater oil sorbate absorptivity ranging between 77 and 104%. These values indicate that NSF has a greater affinity for oil than water, encompassing different oils with varying properties and nature, from vegetable-based to petroleum-derived oils. Interestingly, it can be further noted that the general trend of the sorption capacity of the foam is roughly correlated with the viscosity of the oil, as seen in Table 1. This is because as the viscosity increases, the high-viscosity oils tend to cling more to the cell wall of the sorbent material, whereas low-viscosity oils are easily liberated from the sorbent material during the draining stage^13^.

**Table 1:** Summary of viscosities and densities of the different sorbates used in this study.

	Density (g/cm^3^)	Viscosity (Pa s)
Distilled water	1.00	1.00 × 10^−3^
Seawater	1.03	9.20 × 10^−4^
Vegetable oil	0.91	99.60
Engine oil	0.83	494.00
Used engine oil	0.91	413.33
Bunker fuel	0.99	1986.67

### Foam recyclability

**Figure 7:**
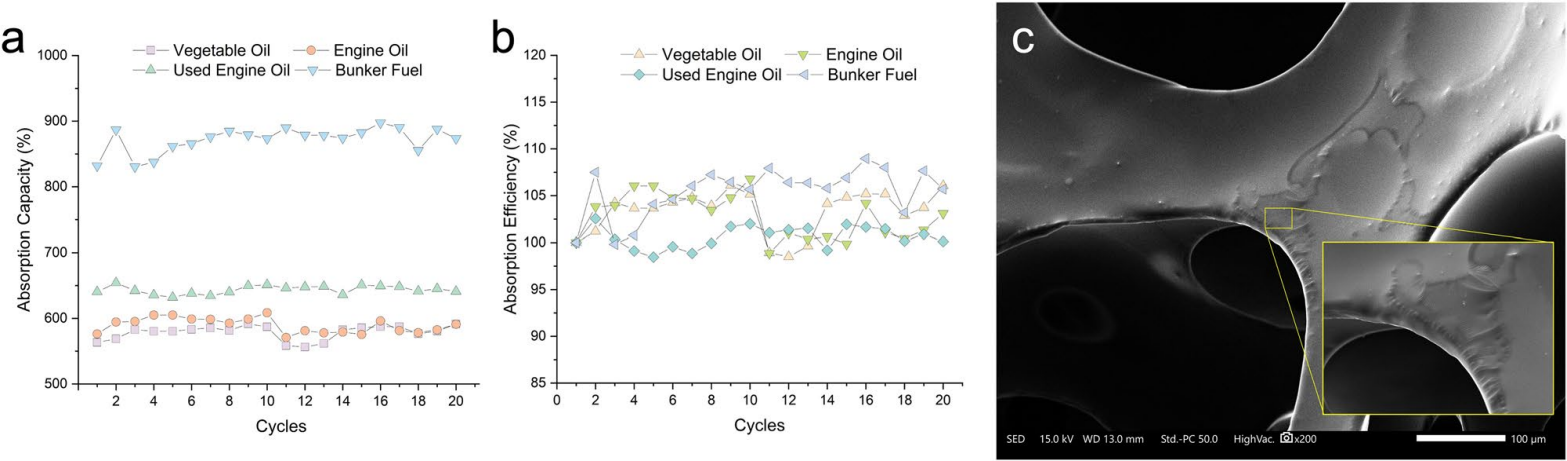
The absorption (**a**) capacity and (**b**) efficiency of the naturally superoleophilic foam (NSF) after 20 cycles and (**c**) the SEM microstructure of spent NSF showing the swelling of cell struts after multiple uses.

The determination of the recyclability of NSF plays a pivotal role in its practical application for oil spill clean-up efforts. Thus, the NSF samples were subjected to a series of sorption–desorption cycles, and the sorption capacity and efficiency (%) per cycle illustrated in Fig. 7a and b were calculated based on the initial weight of the foam. The NSF demonstrated a relatively consistent sorption capacity throughout the 20 continuous sorption–desorption cycles. It can be observed that the sorption capacity at the beginning of the recyclability test is almost similar to the results presented in the static sorption test. However, as the cycles proceed, a slight increase in the capacity becomes apparent. This phenomenon^4^ is presumably ascribed to some restrictions^40^ in the passage of sorbate through the cells at the beginning of the sorption–desorption cycle due to the presence of cell membrane remnants on the open cells as well as the remaining partially closed cells in the foam, making these regions partly impervious to the sorbate, especially for highly viscous oils. As the cycles progress, the repeated swelling of the cells, as seen in Fig. 7c, due to sorbate uptake and the desorption mechanism exert pressure on these weak areas that eventually buckle and make the foam matrix more available for sorption, thus marginally increasing the capacity.

Figure 7b depicts the absorption efficiency of the foam after 20 cycles. From the image, the oil sorption efficiency of NSF remained relatively constant throughout the cycles, implying that it could be recycled without a significant decline in its absorption performance. The fluctuation in the efficiency of the foam is attributed mainly to the residual oils in the foam that were not completely liberated during the desorption process, therefore reducing the sorption capacity of the NSF. These results reveal that the synthesized NSF has good recyclability performance, showcasing its ideal potential in practical oil spill applications.

**Figure 8:**
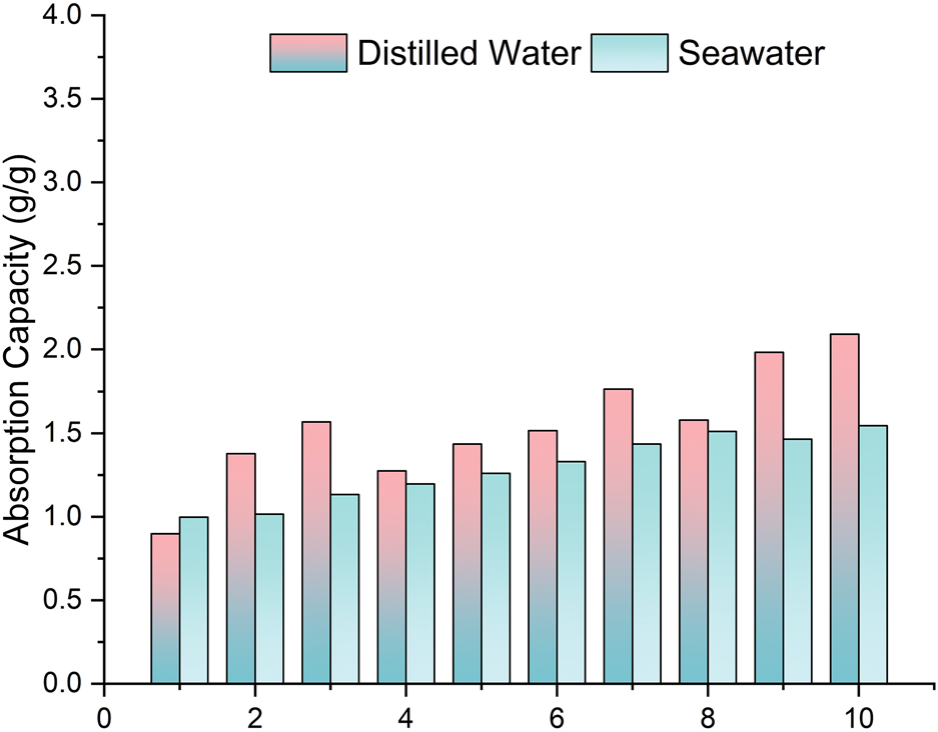
Water sorption capacity of the naturally superoleophilic foam (NSF).

The water sorption capacity of the NSF was also tested for 10 cycles. The results in Fig. 8 show that as the number of cycles increases, the water sorption capacity of the foam also gradually increases. However, this increase is only minimal, accounting for a 1.5–2 times increase in the initial water sorption capacity of NSF. It is also evident in the illustration that the sorption capacity of the foam is slightly higher for distilled water than seawater. This behavior is also observed in other studies^41^ and is attributed to the higher average surface tension of seawater than distilled water, which influences the adhesion and wetting interactions between these sorbates and NSF^42^.

### Stability of the synthesized foam

**Figure 9:**
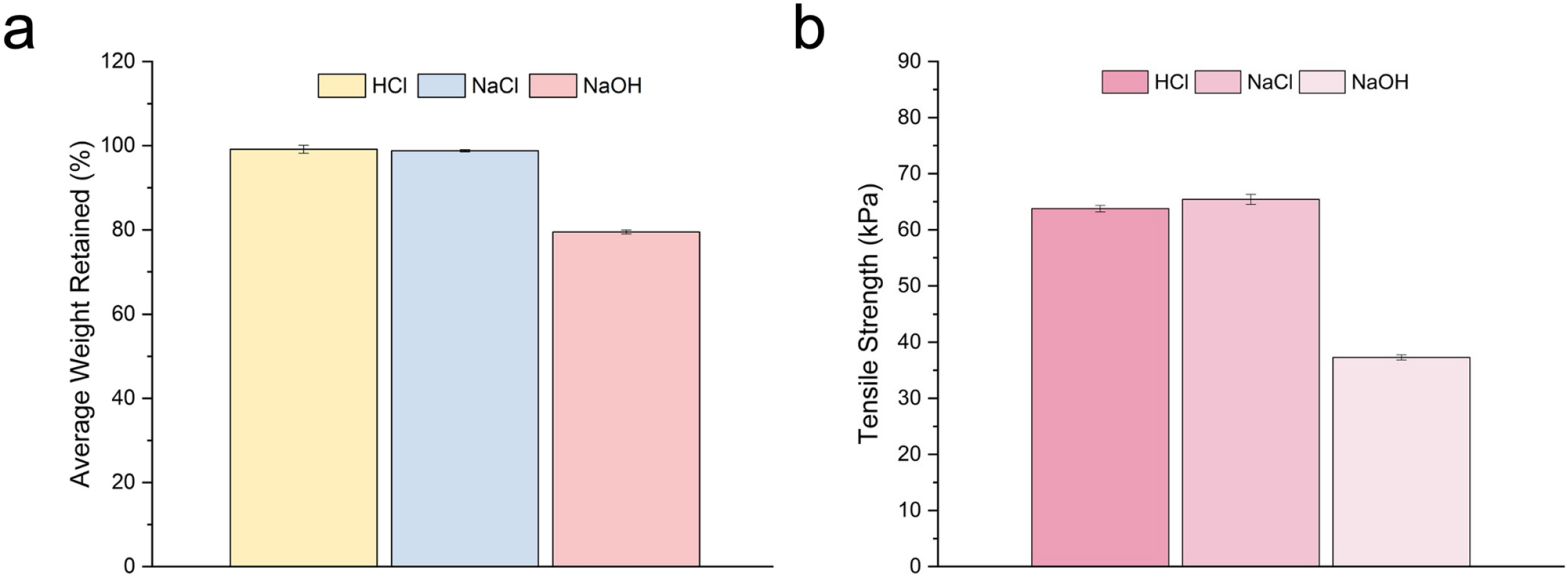
The (**a**) corresponding weight changes and (**b**) tensile strength of the naturally superoleophilic foam (NSF) after soaking in acidic, saline, and basic environments for 24 h.

**Figure 10:**
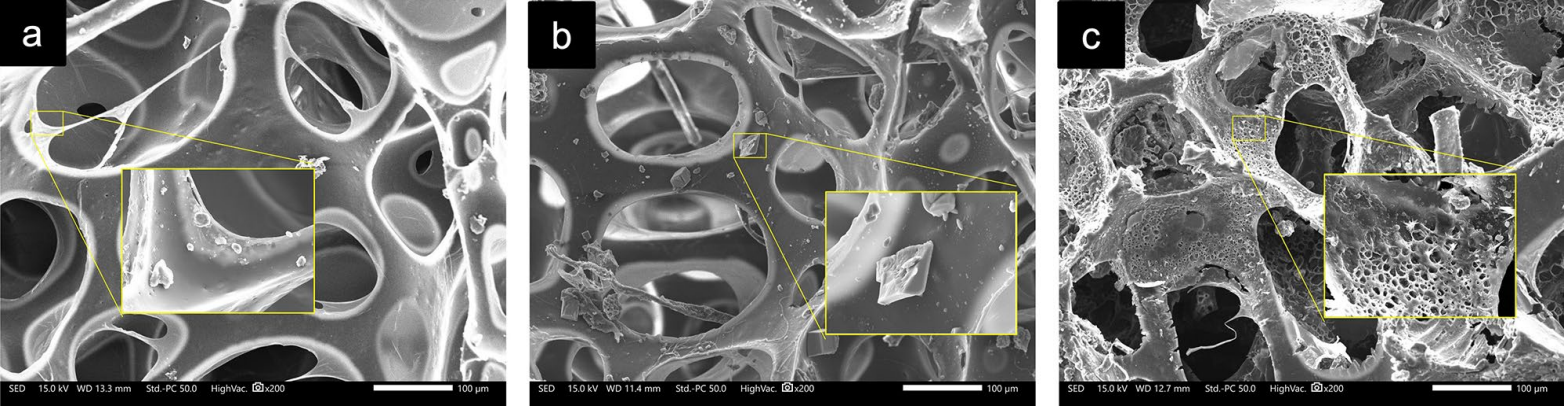
SEM micrographs of the naturally superoleophilic foam (NSF) after a 24-h soaking in (**a**) acidic (HCl), (**b**) saline (NaCl), and (**c**) basic (NaOH) environments.

To demonstrate the chemical stability and durability of the foam against a highly complex environment with various acidic, saline, and basic conditions, the NSF was completely immersed in solutions of 2 M HCl, concentrated NaCl, and 2 M NaOH for 24 h. These tests evaluate the material’s response to these conditions that encompass the extremities of the pH spectrum in terms of changes in weight, strength, and structure as a measure of its chemical resistance. Results of NSF’s chemical stability are shown in Figs. 9 and 10.

Figure 9a depicts the chemical resistance of NSF in reference to its weight changes after being subjected to acidic, saline, and basic solutions. It can be observed from the graph that only minimal weight reduction was recorded for the sample in acidic and saline solutions, signifying excellent stability in these environments. However, a substantial reduction was recorded under basic conditions. Moreover, the influence of the different chemical conditions on the mechanical strength of NSF was evaluated through its tensile performance after the test. This is in relation to the potentially huge impact that the previously reported changes in the material’s weight have on its tensile strength. Remarkably, the NSF soaked in acid and saline solutions in Fig. 9b revealed an increase in tensile strength, amounting to 17.6 and 20.7% relative to the initial value in Fig. 4. On the other hand, a 31.2% decrease was detected for the foam subjected to the basic reagent.

In addition, the structural changes effected by the chemical stability test on the NSF were also probed, and the resulting micrographs of the foam are shown in Fig. 10. It was evident from the images in Fig. 10a and b that the acid and saline solutions did not cause any undesirable modifications on the foam other than the minute amount of HCl and NaCl crystals deposited on the foam surface. Comparatively, structural damage and deformation can be seen on the SEM image of NSF in Fig. 10c, with apparent strut thinning and breakage, showing a visual account of the weight and strength reduction of the foam. A greater degree of NaOH crystal deposition is also noted in the sample. These observations on the weight, strength, and structural changes that the NSF underwent as a result of the chemical testing impart a particularly concurring outcome that exemplifies the overall chemical resistance of the material. Based on empirical data, the NSF possesses high resistance and stability in acidic and saline conditions. The same cannot be said in basic conditions, however, as the NSF showed a significant decrease in weight and strength, as well as disruption in its structure. This behavior may be owed to the susceptibility of the ester groups in the polyol and the urethane linkages in the foam matrix to hydrolytic degradation in highly basic solutions^43,44^.

## Conclusion

This study introduces a novel naturally superoleophilic PU foam with hydrophobic properties, synthesized via the conventional one-shot foaming method with the incorporation of a bio-based polyol derived from coconut oil for oil spill remediation. The foam exhibits an inherently high oil sorption capacity of up to 24.65 g g^−1^, strong affinity to various types of oil contaminants, including heavy oils, excellent hydrophobic nature with a water contact angle of 138.7°, excellent reusability with only a minimal decline in sorption capacity after 20 cycles, and exceptional stability in extremely acidic, saline, and basic environments. These findings highlight the promising potential of this sorbent for practical oil removal applications, underscoring its facile production, marking a transformative shift in a greener approach and meaningful progress in mitigating the impacts of oil spills.

## Data Availability

Data will be made available upon reasonable request. Correspondence and requests should be addressed to the primary corresponding author, A.A.L.
